# Smoking cessation treatment prior to psychotherapy for patients with diagnosed mental disorders: study protocol for a randomized controlled trial

**DOI:** 10.1186/s13063-025-08917-4

**Published:** 2025-07-01

**Authors:** Esra Teresa Sünkel, Marie Neubert, Alla Machulska, Tim Klucken

**Affiliations:** Department of Clinical Psychology and Psychotherapy, University of Siegen, Obergraben 23, Siegen, 57072 Germany

**Keywords:** Smoking cessation, Tobacco dependence, Cognitive behavioral therapy, Short-term treatment, Substance use disorders

## Abstract

**Background:**

Tobacco use is a leading risk factor for premature mortality. Individuals with mental disorders exhibit a smoking prevalence twice that of the general population and engage in higher levels of tobacco consumption, thereby elevating their risk for tobacco-related health complications. Unlike with other substance use disorders, clinical practice in Germany neglects tobacco dependence as a risk factor prior to the initiation of psychotherapy, despite a clear need for intervention: Prolonged cigarette smoking exacerbates mental health symptoms and influences processes central to psychotherapy, such as implicit cognitive processes and emotion regulation. Moreover, short-term nicotine withdrawal associated with tobacco dependence can undermine motivation, reduce positive reinforcement, and cause emotional instability, potentially hindering efforts to improve mental health or the effectiveness of psychotherapeutic interventions. These factors underscore the importance of integrating smoking cessation strategies prior to formal psychotherapy to optimize the therapeutic process and patient outcomes.

This study investigates the effects of initiating an intensified smoking cessation program prior to starting psychotherapy. Main measure outcomes of interest are the degree of tobacco dependence, smoking behavior, and mental health outcomes in smokers with mental disorders. By addressing smoking behavior prior to therapy, this trial seeks to establish a more solid foundation for therapeutic work, potentially improving mental health outcomes and promoting a comprehensive approach to mental health care.

**Methods:**

A single-center randomized controlled trial in an outpatient psychotherapeutic clinic will examine the effects of an intensified smoking cessation intervention versus a waiting control condition. The primary outcome measures include smokers’ nicotine dependence, smoking behavior, and mental health symptoms related to their primary mental disorder, analyzed using variance analysis methods. The smoking intervention consists of a common guideline-based cognitive-behavioral program supplemented by a digital health application. Secondary outcomes include changes in self-reported self-efficacy and implicit cognitive biases. In addition, motivational measures, self-efficacy, implicit approach tendencies, and attitudes toward online interventions will be assessed as secondary measures and examined as potential predictors, moderators, or mediators in exploratory analyses. Attitudes toward online interventions will be measured once at baseline, while all other measures will be assessed before the intervention, post-treatment, and at 6-week and 6-month follow-up sessions.

**Discussion:**

Addressing smoking behavior before psychotherapy holds potential to significantly enhance mental health outcomes. This study investigates the integration of smoking cessation strategies prior to psychotherapeutic care for cigarette-smoking individuals with mental disorders. By aligning tobacco dependence treatment with approaches used for other substance use disorders, we aim to establish and discuss a comprehensive and guideline-conformant method. This strategy seeks to improve patient outcomes and a cohesive, integrated method for treating smokers with comorbid mental health disorders.

**Trial registration:**

Prospectively registered on ISCRTN on 01.05.2024, reference number ISRCTN12859609.

## Introduction

### Background and rationale {6a}

Tobacco use, recognized as the leading cause of preventable death worldwide [38, 73], presents a significant health risk to all, particularly to individuals with mental disorders due to their high smoking prevalence and consumption pattern. These individuals are nearly twice as likely to smoke as the general population [5, 59], often smoking more heavily [10] and relying on tobacco to cope with negative affect, rather than to gain pleasure [64]. This increased severity of tobacco use contributes to a drastically reduced life expectancy—up to 25 years shorter—for smokers with mental disorders compared to non-smoking individuals without mental disorders [13, 30, 44]. According to the World Health Organization [72], cigarette smoking remains the most common form of tobacco use. Notably, clinical practice for treating tobacco dependence among the population of smokers with mental disorders deviates from that for other substance use disorders. Despite the clear need for intervention, smoking cessation in individuals with comorbid mental disorders remains challenging, as evidenced by their persistently high smoking prevalence [5, 70].

The need for intervention in this specific population not only stems from proven fatal physical consequences resulting from prolonged tobacco consumption, but also from psychological and mental consequences. Nicotine, the primary alkaloid in tobacco responsible for the addictive potential of cigarette smoking, poses significant hazards to psychological well-being, making it particularly relevant in the treatment of smokers with mental disorders. When initiating psychotherapy, the professional code of conduct for psychotherapists in Germany mandates prioritizing abstinence within the initial ten sessions if a patient meets diagnostic criteria for substance dependence or abuse [62]. However, tobacco dependence or abuse is excluded from these regulations. This is surprising given the existing research on nicotine’s impact on mental health symptoms as well as evidence highlighting nicotine’s influence on neuropsychological processes. Looking at nicotine’s impact on mental health, research shows associations of smoking and a decline in mental health symptoms, such as reduced quality of life [56], poor sleep quality [31], and higher rates of depressive and anxiety symptoms [12, 25, 48]. Among smokers with mental disorders, tobacco consumption may exacerbate mental health symptoms, while psychological distress can intensify smoking, creating a vicious cycle. Recent meta-analyses have indicated that successful tobacco cessation has long-term positive effects on mental health, as indicated by reduced symptoms of depression and anxiety, as well as overall increased quality of life [14, 63], and that these positive effects are comparable or even superior to those following pharmacological antidepressant treatment [63].

Nicotine withdrawal, which occurs not only during deliberate cessation, but also is experienced by regular smokers during intervals when nicotine levels decline due to its short half-life, and smoking is not immediately feasible, further complicates the picture by negatively affecting cognitive and emotional processes. Nicotine withdrawal is linked to memory deficits [55], concentration difficulties and increased impatience [28], and impulsivity and impaired decision-making [35]. Such cognitive impairments can hinder the patient's engagement with cognitive-behavioral techniques, emphasizing the need to address smoking before psychotherapy begins. Additionally, nicotine withdrawal reduced neurobiological reward sensitivity with diminished responses to positive stimuli persisting for several days [19, 35]. In the context of patients aiming to initiate psychotherapy, a diminished reward system due to prolonged tobacco smoking may reduce the motivation and positive reinforcement essential for behavioral change. Furthermore, the cyclical nature of withdrawal can cause emotional dysregulation and mood instability [43], potentially reducing therapy adherence and overall treatment engagement.

Building on the understanding of how nicotine withdrawal impairs cognitive and emotional functioning, it is also crucial to consider the broader neuropsychological effects of nicotine use, particularly in the context of patients aiming to undergo psychotherapy. Research suggests that nicotine, like other psychoactive substances such as alcohol, can significantly affect learning processes relevant to psychotherapy. So-called dual-process models of addiction (e.g. [68]) provide a useful framework for understanding these processes. According to these models, tobacco dependence can be described as a result of interactions between a reflective system and an impulsive system. The reflective system encompasses deliberate, goal-directed decision-making and self-regulation, while the impulsive system—driven by associative learning and emotional responses—manages automatic cravings triggered by stimuli and conditioned responses from repeated nicotine use, often referred to as implicit processes [17]. Implicit processes play a crucial role in maintaining tobacco dependence. Research supports this model by demonstrating that prolonged tobacco consumption is linked to cognitive biases associated with these implicit processes. For example, prolonged tobacco use is associated with attentional biases towards smoking-related cues [22], approach biases (e.g., [41, 67]), and interpretation biases [40]. Taken together, current research shows that prolonged tobacco use is linked to declines in mental health, cognitive and emotional impairments, and the development of cognitive biases and automatic action tendencies. Integrating smoking cessation strategies prior to psychotherapeutic treatment could thus enhance mental health outcomes and increase patient receptivity by mitigating nicotine-related neuropsychological disruptions that undermine the effectiveness of interventions dependent on cognitive and emotional engagement.

Despite this clear benefit, however, clinical practice in Germany still faces significant challenges in effectively addressing tobacco dependence among patients with comorbid mental disorders [5]. The national Association of the Scientific Medical Societies in Germany (AWMF) guideline for tobacco-related disorders [5] recommends that smokers with comorbid mental disorders receive the same smoking cessation interventions as those offered to the general population. These include pharmacological treatments (for an overview see [28]), cognitive–behavioral therapy (CBT)-based interventions (e.g., [37]), smartphone-based self-help programs (e.g., [54]), or multimethod approaches (e.g., [24]), with clinical consensus indicating that more complex and intensive smoking cessation programs may yield better outcomes and long-term abstinence for smokers with comorbid mental disorders [45].

In summary, research consistently highlights the detrimental effects of smoking on mental health, particularly on emotional, cognitive, and implicit processes, which is especially relevant in the context of psychotherapy and a holistic approach to mental health. At the same time, there is a notable gap in addressing tobacco dependence among psychotherapeutic patients [5]. In light of these gaps, the current study seeks to investigate the effects of an intensified smoking cessation program for smokers with mental disorders, implemented prior to starting formal psychotherapy. By this, our study seeks to align the standard treatment of tobacco dependence with the approach taken for other substance use disorders within the context of psychotherapeutic care in Germany. By targeting smoking behavior before the commencement of psychotherapy, we aim to establish a more solid foundation for therapeutic work, improving patient outcomes prior to the initiation of psychotherapy for the treatment of the primary mental disorder.

To this end, we employ a multifaceted smoking cessation intervention that combines face-to-face sessions with licensed psychologists and an additional digital health application. We will evaluate the effects of this approach on tobacco dependence, smoking behavior, and mental health outcomes related to the primary mental disorder. Additionally, the study will examine predicting, moderating, or mediating factors such as motivation to quit, willingness to change, self-efficacy, and attitudes towards online interventions to understand their influence on treatment and inform secondary analyses. To integrate both reflective and implicit cognitive processes, we are also incorporating the Approach-Avoidance Task (AAT; [53]) as an additional measure for implicit processes associated with tobacco dependence. The task has been proven effective in assessing and altering drug-related approach biases for substances like alcohol [69], cannabis [15] and recently, for tobacco [39, 42, 61]. By incorporating the AAT in our study, we aim to measure implicit cognitive biases related to smoking and how they change in response to smoking cessation treatment, thereby advancing the understanding of how addressing smoking prior to psychotherapy affects patients. By integrating smoking cessation into standard psychotherapeutic care, we aim to offer new insights into its potential benefits and emphasize the importance of incorporating smoking cessation as a critical component of comprehensive mental health treatment.

### Objectives {7}

We expect a treatment effect of the intensified smoking cessation on variables regarding tobacco dependence severity, smoking behavior as well as mental health outcomes, as evidenced by a reduced nicotine dependence severity, reduced smoking intensity, a higher quality of life and subjective psychological well-being, and reduced perceived impairment of mental health symptoms and depressive symptoms in patients in the experimental condition receiving a smoking cessation intervention than in patients in the wait-list-control condition. These changes are expected to become apparent both in the short-term (i.e., post-treatment) as well as the long-term (i.e., 6 weeks and 6 months follow-up) when compared to the baseline measures.

### Trial design {8}

The present research project demonstrates a single-center randomized controlled trial examining the impact of an intensive smoking cessation treatment compared to a waiting control condition on tobacco dependence and mental health symptoms related to the primary mental disorder among smokers aiming to initiate outpatient psychotherapy. All participants included in the study are receiving three psychotherapeutic consultations and fulfill an indication for psychotherapy. We chose to include a waiting control condition instead of an active (unspecific) control condition for comparison as a waiting control condition mirrors the typical course for patients awaiting psychotherapy, making the findings more applicable to real-world clinical settings. Eligible participants will be randomly assigned to either the experimental condition (immediate diagnostic session, a six-session smoking cessation program and a self-guided digital health application) or the waiting control condition (diagnostic sessions only). Patients randomly assigned to the control condition will be placed on a wait-list and will receive the smoking cessation intervention after 6 months. Post-treatment assessments for the intervention group will occur three weeks after their initial diagnostic session, which marks the end of the treatment phase. Similarly, participants in the control condition will undergo post assessments 3 weeks after their initial diagnostic session. Follow-up assessments for both groups will take place at 6 weeks (follow-up 1) and 6 months (follow-up 2) after the completion of the post-treatment assessments.

## Methods: participants, interventions, and outcomes

### Study setting {9}

The study will take place at the University of Siegen’s outpatient psychotherapeutic clinic.

### Eligibility criteria {10}

Participants must be at least 18 years old and exhibit harmful use of tobacco (F17.1 according to the ICD-10; [47]) or tobacco dependence (F17.2 according to the ICD-10) while also fulfilling the criteria for a treatment-relevant primary mental disorder. An exclusion criterion is acute suicidality, as well as the presence of an acute psychotic episode or the use of certain medications (e.g., neuroleptics such as Clozapine), where unmonitored cessation without a psychiatrist’s oversight may pose significant risks. The smoking intervention will be performed by trained clinical psychologists, while all psychotherapeutic consultations will be performed by trained psychological psychotherapists. If additional substance use disorders are present, treatment of these disorders is prioritized in accordance with German clinical guidelines [62] and ethical standards, particularly when inpatient treatment is indicated. Smoking cessation is then offered as an adjunctive treatment once the primary substance-related condition is overcome. This approach ensures appropriate care and participant safety.

### Who will take informed consent? {26a}

The informed consent of study participants is ensured through a comprehensive briefing at the initiation of the study (initial session) by the clinical psychologist. After receiving and reviewing the information, participants’ consent will be obtained.

### Additional consent provisions for collection and use of participant data and biological specimens {26b}

Not applicable.

## Interventions

### Explanation for the choice of comparators {6b}

By comparing the intensified smoking cessation program implemented prior to psychotherapy to a waiting control condition, we aim to evaluate the effectiveness and potential superiority of our approach regarding smoking cessation success and improvement in mental health symptoms. It is important to acknowledge that participants in both conditions may seek or receive other forms of psychotherapeutic treatment during the post- and 6-month follow-up assessments. To account for this, we will inquire about any additional treatments participants may have undergone and control for these variables in our analysis. Specifically, all additional treatments will be systematically documented and coded as categorical variables (e.g., concurrent individual psychotherapy, group therapy, psychopharmacological treatment). Dummy variables representing the presence or absence of each treatment type will be included as covariates in the repeated-measures ANOVAs. This approach not only ensures a more accurate comparison but also reflects the naturalistic conditions under which the study is conducted.

### Intervention description {11a}

The intervention for the experimental group consists of a manual-based six-step smoking cessation program, adapted from the widely implemented and continuously evaluated smoking cessation program developed by the *Arbeitskreis Tabakentwöhnung* (Smoking Cessation Working Group) at the University Hospital for Psychiatry and Psychotherapy in Tübingen. This program, detailed in the manual by Batra and Buchkremer [3] and recently updated [4], is rooted in cognitive–behavioral therapy (CBT) and designed for both group and individual settings, though this study will utilize the individual setting. The program spans six sessions, during which participants will receive guidance on preparing for and executing their smoking cessation efforts, developing strategies to maintain abstinence, and preventing relapse. The sessions are organized into three distinct phases, as outlined in Table 1.

**Table 1:** Smoking intervention phases in the intervention group

Phase 1	Session 1	Abstinence preparation phase: Focuses on motivating individuals and understanding their smoking habits
	Session 2	
Phase 2	Session 3	Cessation phase: Provides specific strategies for quitting smoking and managing cravings
	Session 4	
Phase 3	Session 5	Stabilization phase: Emphasizes building alternative behaviors, coping with potential relapse triggers, promoting overall health, and managing relapses
	Session 6	

Additionally, personalized recommendations tailored to each individual’s smoking behavior will be provided. Detailed descriptions of the individual sessions are outlined in the manual by Batra and Buchkremer [4]. In this research project, we will augment the original program by increasing session frequency and incorporating a self-help smoking cessation smartphone application to address specific challenges faced by smokers with mental disorders. While the original program was designed with weekly sessions over a course of 6 weeks, our intensified version will include two sessions per week, shortening the intervention duration to three weeks. Furthermore, participants will have access to the digital health application (*NichtraucherHelden®*,Sanero Medical GmbH, Stuttgart, Germany), a CBT-based self-help app featuring eight modules of psychoeducation, tasks, and relapse prevention. Recent evidence from a nationwide, multicentric, parallel, randomized controlled trial demonstrated the app’s feasibility and efficacy, with the intervention condition showing twice the abstinence rates at a 7-day point prevalence compared to the control condition [54].

### Criteria for discontinuing or modifying allocated interventions {11b}

The assigned intervention can be terminated upon the patient's request, and participants may withdraw from the study for any reason. Researchers retain the authority to discontinue a patient's participation in the study if their mental health condition worsens, such as through experiencing a psychotic episode, exhibiting an acute increase in suicide risk, or an indication for hospitalization.

### Strategies to improve adherence to interventions {11c}

Protocol adherence is monitored by the authors of the study, who ensure that the manual-based intervention is conducted as intended and that all the data were collected as described. To support and enhance adherence, all interventionists undergo training to deliver the intervention, emphasizing the importance of fidelity to the manual. After each session, a session protocol is created in which any deviations from the manual or noteworthy occurrences are documented. This ongoing documentation allows for timely feedback and corrective actions if necessary, further reinforcing adherence to the intervention protocol.

### Relevant concomitant care permitted or prohibited during the trial {11d}

There is no specific concomitant care administered nor prohibited during the trial.

### Provisions for post-trial care {30}

There is no anticipated harm or compensation for trial participation.

### Outcomes {12}

Primary outcomes include severity of tobacco dependence, smoking status, and mental health symptoms. All measures will be assessed at baseline (diagnostic session; *t*_0_), post (*t*_1_; *t*_0_ + 3 weeks), at follow-up assessment 1 (*t*_2_; *t*_1_ + 6 weeks), and at follow-up 2 (*t*_3_; *t*_1_ + 6 months).

#### Primary outcomes targeting smoking behavior

Changes in tobacco dependency over time are operationalized as follows:

- Mean score changes in the"Fagerström Test for Nicotine Dependence"[20],German version: [9].
- Mean score changes in the “Alcohol, Smoking, and Substance Involvement Screening Test” (ASSIST) subscale for tobacco [58].
- Mean score changes in the measured carbon monoxide (CO) level in exhaled breath using the Smokerlyzer®, additionally assessed during each session of the intervention.

Changes in smoking status over time are operationalized as follows:

d. Changes in the proportion of positive versus negative results in self-administered urine tests measuring cotinine content, using a dichotomous outcome (positive/negative; cutoff 200 ng/ml; for an overview, see [8]).

#### Primary outcomes targeting mental health symptoms

Changes in mental health symptoms related to mental disorders are measured by self-report questionnaires, which will also be assessed at the four measurement points mentioned above (baseline, post, follow-up 1 and 2). The questionnaires target general psychological well-being and perceived quality of life, symptoms associated with mental disorders, and depressive symptoms.

Changes in mental health status over time are operationalized as follows:

- Mean score changes in the German version of the “WHO-5 Well-being Index” ([WHO-5]; [52]).
- Mean score changes in the German version of the “WHO Quality of Life-BREF” ([WHOQOL-BREF]; [1]).
- Mean score changes in the “Brief Symptom Check List” ([BSCL]; [23])
- Mean score changes in the “Beck Depression Inventory-Revised” ([BDI-ll]; [6]).

#### Secondary and exploratory analyses

Secondary and exploratory analyses will investigate the impact of the treatment on other clinically relevant factors, such as implicit processes associated with smoking, alongside examining potential moderators or mediators, such as both global and smoking-related self-efficacy, both global motivation to change and specific motivation to quit smoking, and attitudes towards online interventions. All secondary variables will be assessed at all measurement points (baseline, post, follow-up1 and 2), with the exception of attitudes towards online interventions, which will only be assessed at baseline.

##### Implicit processes

Changes in implicit processes associated with smoking over time are operationalized as follows:

- Mean score changes in the nicotine-related approach biases as assessed with the Approach-Avoidance Task [53] adapted for smoking [42].

##### Self-efficacy

To capture a comprehensive view of self-efficacy, self-efficacy is assessed by two questionnaires. This dual approach ensures that both broad and behavior-specific aspects of self-efficacy are captured. We will examine changes in both general and smoking-related self-efficacy as potential moderating or mediating factors of the treatment effect, as well as secondary outcome variables.

Changes in both general and smoking-related self-efficacy are operationalized as follows:

b. Mean score changes in the German version of the “Generalized Self-Efficacy Scale” ([GSE]; [33]).
c. Mean score changes in the German version of the “Self-Efficacy Scale for Smoking” ([SESS]; [32]).

##### Motivation

Changes in patients’ motivation to change will be assessed using multiple questionnaires to provide a detailed view of their readiness for change, stage of behavioral change, and specific motivation to quit smoking. By employing a range of measures, we aim to capture various dimensions of motivation and how they evolve throughout the intervention. This approach will allow us to analyze changes in motivation as potential moderating or mediating factors in the treatment effect, and to better understand the role of motivation in the overall effectiveness of the smoking cessation program.

Changes in patients’ motivation are operation as follows:

d. Mean score changes in the German version of the “University of Rhode Island Change Assessment Scale” ([FEVER]; [26]).
e. Mean score changes in the “Scale for Stages of Change” ([SOC]; [18]).
f. Mean score changes in the “Motivation to Stop Scale” ([MTSS]; [49]).

##### Attitudes toward digital health applications

Patients’ attitudes towards online interventions are measured as follows:

g. Mean scores of the questionnaire “Attitudes toward Psychological Online Interventions–the APOI” ([APOI]; [57]).

### Participant timeline {13}

Smoking patients who have had three psychotherapeutic consultations at the Psychotherapeutic Outpatient Center of the University of Siegen will be informed about the option of participation in the current study. Interested participants will be contacted by the study team and undergo a telephone interview to determine eligibility and fulfillment of the inclusion criteria (*− t*_1_). Following telephone screening for a duration of 10–30 min, patients will be invited to the first smoking cessation session, during which informed consent will be obtained and psychological symptoms will be assessed within the course of a diagnostic session (baseline, *t*_*0*_). After the diagnostic session, during which all baseline measures will be assessed, patients will be randomly assigned either to the experimental group or the passive control group. The experimental condition will receive a 6-step smoking cessation program and additional access to a smartphone-based self-help application. The duration of the intervention will be three weeks. Afterwards, patients will be invited again to assess all measures of interest (*t*_1_). Six weeks (*t*_2_) and 6 months (*t*_3_) after completion, patients will be invited once again for follow-up assessments. The participants’ timelines and data assessment points are shown in Table 2..

**Table 2.**
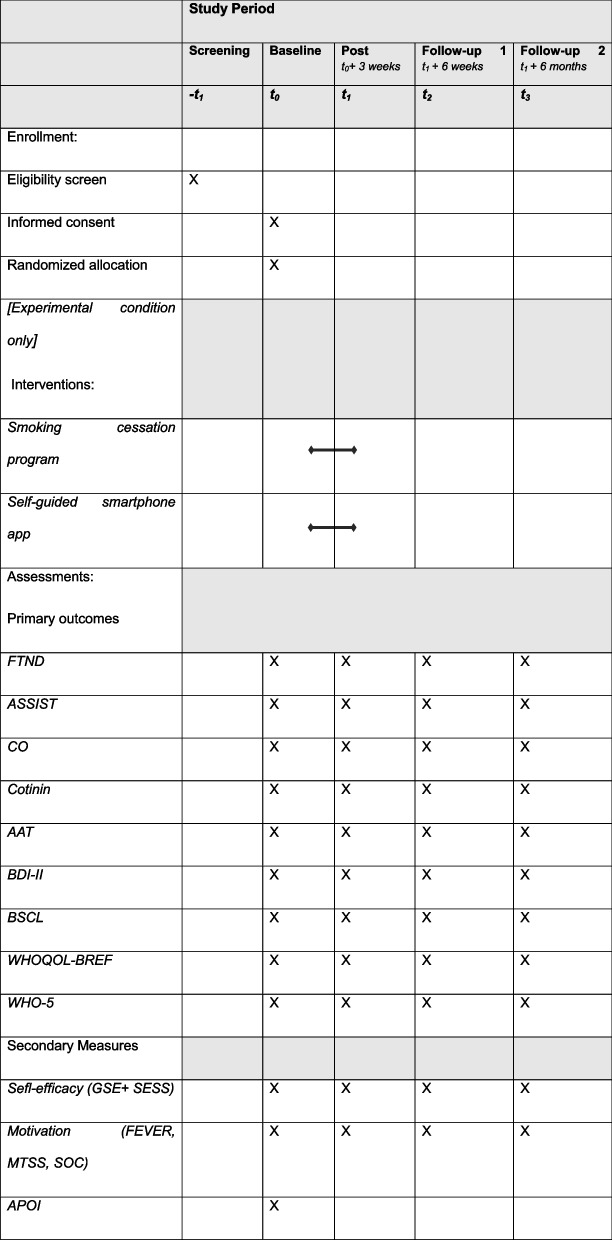
Participant timeline

### Sample size {14}

To estimate the required sample size, an a priori power analysis was conducted using G*Power 3.1.9.7 [21]. The primary hypothesis will be tested using a 2 × 4 mixed-design repeated-measures ANOVA (with a power of 1 − *β* = 0.80 and a significance level of *α* = 0.05. This requires a sample size of approximately 62 participants in total, 31 per condition, to detect a moderate effect. No additional participants will be recruited to compensate for potential dropout; instead, the study design follows the intention-to-treat (ITT) principle and employs multiple imputation methods to address missing data. The decision to target a moderate effect size is driven by practical considerations to detect treatment effects that are both statistically significant and practically meaningful.

### Recruitment {15}

Participants will be recruited among individuals identified as regular smokers who previously had up to three psychotherapeutic consultations in the outpatient psychotherapeutic clinic at the University of Siegen. If the inclusion rate does not meet the requirements, additional advertisements to enhance participation in this project will be installed by the research team. If necessary, the planned duration of the enrollment period of 1 year will be extended.

## Assignment of interventions: allocation

### Sequence generation {16a}

Participants will undergo random allocation to either the experimental or control condition at a 1:1 ratio. Given the study design, participant or therapist blinding post-allocation is not feasible.

### Concealment mechanism {16b}

No concealment mechanism, the groups are randomly allocated.

### Implementation {16c}

A research assistant or psychotherapist will communicate the allocation to patients and introduce them to either the intervention or the control condition.

## Assignment of interventions: blinding

### Who will be blinded {17a}

Not applicable. The nature of the study’s intervention does not allow blinding.

### Procedure for unblinding if needed {17b}

Not applicable. The nature of the study’s intervention does not allow blinding.

## Data collection and management

### Plans for assessment and collection of outcomes {18a}

In this study, training of assessors will be facilitated by the research team to ensure standardized administration of the study instruments. The study instruments encompass a comprehensive array of assessments. The used questionnaires comprise self-report instruments only, and participants will be invited to complete the questionnaires digitally, meaning that all values will be transferred immediately to our database to promote data quality. The quality of the assessment will be ensured by having all participants complete the questionnaires individually during the diagnostic sessions in a quiet room without any distractions. All of the study’s instruments were chosen based on satisfactory reliability and validity.

#### Primary outcome self-report questionnaires

The FTND [20], a ten-item scale with dichotomic items, is considered highly reliable and valid [50], *rtt* = 0.88, α = 0.61). The scores can range from 0 to 10, while scores from 0 to 2 indicate no/very weak tobacco dependence, scores.

The ASSIST [66] is an efficient measure due to its capability for the simultaneous assessment of multiple addictive substances and has undergone rigorous validation [29, 46, 66]. For tobacco consumption, concurrent validity of the original version of the ASSIST was indicated by significant correlations of substance-specific subscale scores of ASSIST and the scores in FTND (*r* = 0.85; [46]). Average test-retest reliability coefficients range from good to excellent depending on the substance class [66]. Reliability was proven good for tobacco (*α* = 0.83; [65]). Scores from 3–7 indicate moderate dependence, and scores of 8–10 indicate very high dependence.

The BDI-II [6] is a self-report questionnaire assessing the severity of depressive symptoms across 21 symptom areas that has good internal consistency (*α* = 0.90–0.93) and its reliability can be considered good (*rtt* = 0.78; [6]). The total score can range from 0 to 63; scores below 9 indicate no evidence of depression, 9–13 suggest minimal depression, 14–19 indicate mild depression, 20 or above suggest moderate depression, and scores of 29 or above suggest severe depression.

The BSCL [23] assesses an individual's perceived impairment due to 53 physical and psychological symptoms, consists of nine subscales, and has been evaluated in numerous studies. The authors report good internal consistencies ranging from 0.71 to 0.85 and test-retest reliabilities ranging from 0.68 to 0.91, depending on the subscale [23].

The short version of the WHO Quality of Life Questionnaire (WHOQOLBREF) assesses the dimensions of physical well-being, psychological well-being, social relationships, and environment, and is considered to have “good to excellent” psychometric properties [60], with internal consistencies ranging from 0.66 to 0.84 [71].

The WHO-5 is a psychometric tool used to assess an individual's overall well-being and mental health and consists of five 6-point items. The German version has good internal consistency (*α* = 0.92) and very good reliability (*rtt* = 0.87; [11]).

#### Primary outcome instruments targeting biomarkers

Carbon monoxide is assessed using a piCO™ Smokerlyzer® (Bedfont Scientific Ltd.). CO levels are objective markers of heavy smoking, and a value of 7.5 ppm in exhaled CO can be considered an appropriate cutoff to differentiate non-smokers from active smokers [2]. Cotinine is assessed using single rapid urine tests (nal von minden gmbH) with a cutoff of 200 ng/mL, which is a commonly used cutoff for detecting nicotine exposure for up to 5 days [34].

### Primary outcome measures measuring implicit processes

Approach biases towards smoking-related stimuli and non-smoking control stimuli are measured using the standard joystick AAT [53] as modified by Machulska et al. [42]. In this task, participants view various images on a computer screen, each slightly tilted either 3° to the left or to the right. Using a Logitech Extreme 3D joystick connected to the computer, participants are instructed to push images rotated to the right and pull images rotated to the left using the joystick. This action causes the images to either shrink or grow in size, depending on the direction of the arm movement. According to Rinck and Becker [53], an approach bias score for a particular picture category (e.g., smoking or control images) is determined by the difference in reaction times (measured in milliseconds, ms) between approach and avoidance movements. Approach biases are calculated for each of the picture categories and across each diagnostic session. A positive difference score indicates that the participant was quicker to approach than to avoid a particular stimulus category, signifying an approach bias. The task includes 50 smoke-related pictures and 50 non-smoking control pictures. Each picture will be presented once in push-away format and once in pull-closer format, respectively, resulting in 200 total trials.

#### Secondary measures: self-report questionnaires

The GSE measures generalized self-efficacy and has good reliability (*α* = 0.71–0.89; [33]). The unidimensional SESS measures smoking-related self-efficacy and consists of 9 items, demonstrating an internal consistency of *α* = 0.95 and test–retest reliability of *r* = 0.85 [32]. The FEVER measures stages of change and has good internal consistency, with Cronbach’s alpha ranging from 0.76 to 0.86 depending on the subscale [26]. For the SOC, a scale also measuring stages of change that varies from 0 (precontemplation) to 4 (maintenance), studies have reported a test–retest product-moment correlation of *r* = 0.78 [18]. The MTSS measures the motivation to quit smoking, and area under the receiver operating characteristics (ROC_AUC_) curves of the MTSS show good external validity (ROC_AUC_ = 0.64, [49]). The authors of the APOI report good internal consistency for depressive patients (*α* = 0.77). In addition, convergent validity was tested by analyzing correlations of the APOI with an established instrument and was proven to be adequate, *r* = 0.74 [57].

### Plans to promote participant retention and complete follow-up {18b}

Participants will be informed about the study’s objective and the importance of completing the treatment and the follow-up evaluation, emphasizing the potential personal benefits of the treatment. Patients will receive a telephone call prior to each follow-up assessment to remind them of the date and encourage adherence to the program.

### Data management {19}

Data entry will be performed under the supervision of the research staff. The data will be electronically stored on a secure research server provided by the University of Siegen.

### Confidentiality {27}

All the data will be collected and stored in a pseudonymized manner (i.e., with a subject code) on a secure, access-restricted research server provided by the University of Siegen. Only anonymized data will be published. Subsequent linking of individuals to their data can only be performed with a datasheet, which will be securely stored and destroyed after the study is completed (but no later than 10 years).

### Plans for collection, laboratory evaluation and storage of biological specimens for genetic or molecular analysis in this trial/future use {33}

Not applicable. Cotinine urine tests will be evaluated immediately after the urine sample is provided and disposed of after using self–diagnostic test strips.

## Statistical methods

### Statistical methods for primary and secondary outcomes {20a}

Treatment effects on the continuous primary outcomes (i.e., tobacco dependence, smoking intensity, mental health symptoms) will be tested by computing mixed 2 (condition) × 4 (time) analyses of variance (ANOVAs) for repeated measurements for each applied outcome variable. To reduce the likelihood of Type I errors due to multiple comparisons, the false discovery rate (FDR) will be controlled using the Benjamini-Hochberg procedure [7] across the primary outcome measures. In the case of potential violations of the sphericity assumption, the Greenhouse–Geisser method will be applied.

It will be examined whether the experimental condition leads to higher abstinence rates at post- and follow-up assessments using chi-squared tests.

Secondary and exploratory analyses testing for potential mediating variables will be conducted using regression analyses or structural equation modeling. Treatment effects on the AAT will be tested by computing mixed 2 (condition) × 4 (picture category) analyses of variance.

### Interim analyses {21b}

No interim analyses or formal stopping rules are planned for this study. The decision is based on the low-risk nature of the intervention, which involves behavioral support without invasive procedures or pharmacological treatments. Additionally, given the naturalistic design, we decided not to conduct interim analyses as they might compromise the integrity of the final data analysis. However, we remain attentive to participant safety and feedback. If any adverse events are reported or significant concerns are raised during participant evaluations (as part of Patient and Public Involvement), appropriate steps, including potential study termination, will be considered and documented accordingly.

### Methods for additional analyses (e.g., subgroup analyses) {20b}

Exploratory and demographic variables will be explored for potential associations with the group condition. If deemed appropriate, analyses regarding treatment effects on primary and secondary outcomes will be re-evaluated to control for potential confounding effects.

### Methods in analysis to handle protocol non-adherence and any statistical methods to handle missing data {20c}

Multiple imputation will be used to address missing data for intention-to-treat (ITT) analyses, as ITT prevents biases in the treatment effect [27]. Specifically, multiple imputation by chained equations will be applied under the assumption that data are missing at random. The imputation model will include baseline scores, group assignment, and other relevant predictors of missingness. In addition to the ITT analyses, exploratory per-protocol analyses may be conducted to gain further insight into the effects among participants who completed the study as intended.

### Plans to give access to the full protocol, participant-level data, and statistical code {31c}

The full study protocol, statistical code, and dataset will be available from the corresponding author upon request.

## Oversight and monitoring

### Composition of the coordinating centre and trial steering committee {5d}

The trial is coordinated by the core author group, consisting of the corresponding author and co-authors. Three student research assistants provide support for day-to-day tasks, including participant communication, administrative duties, and data maintenance. The core author group will meet weekly to oversee the trial process. During these meetings, the core author group will discuss the study progress, address challenges, and ensure smooth trial conduct. On a quarterly basis, a current status report will be submitted to the relevant stakeholder group, detailing the status of the project, its progress, and any problems that might have arisen. The stakeholder group comprises ten licensed psychotherapists who do not conduct the smoking cessation. As confirmed through the University of Siegen’s Ethics committee, the trial can be defined as moderate scale and of low-risk nature. Therefore, it was decided not to form a formal Trial Steering Committee. The trial is funded by the University of Siegen’s research unit; there is no other funding party involved.

As part of Patient and Public Involvement, psychotherapists actively address patients'willingness to participate in the study as part of routine care, ensuring that the study design aligns with participants'interests and preferences from the outset. Prior to the study, some patients had expressed a desire for greater support in achieving smoking abstinence within psychotherapeutic care, which was incorporated into the study concept to increase acceptability and relevance. Additionally, participants are encouraged to provide feedback throughout the study, as part of the Patient and Public Involvement process.

### Composition of the data monitoring committee, its role and reporting structure {21a}

No data monitoring committee has been deemed necessary, as this trial is classified as a low-risk intervention.

### Adverse event reporting and harms {22}

Nicotine withdrawal associated with smoking cessation can, in some cases, lead to increased irritability, sleep disturbances, concentration problems, or heightened feelings of hunger. These withdrawal symptoms typically occur in the first few days after smoking cessation and usually subside within 2 to 3 weeks [16]. These topics will be discussed during the smoking cessation program (sessions 1 and 2), and management strategies will be collaboratively developed for and with the patients. For example, relaxation exercises to cope with irritability, adopting sleep hygiene practices, or providing alternatives to manage increased hunger, such as drinking water, consuming sugar-free gum, or engaging in physical activity, will be addressed. No additional mental strains beyond those typical of quitting smoking are expected as a result of the study. Patients who report adverse events will have the option to undergo evaluation by the research staff and a psychotherapist. Oversight of safety, protocol adherence, study quality, and ethical conduct will be the responsibility of the study coordination unit.

### Frequency and plans for auditing trial conduct {23}

Due to the low-risk nature of the intervention, no formal Data Monitoring Committee is established. Instead, trial conduct is regularly monitored through weekly meetings involving the authors and research assistants to review study progress, address challenges, and ensure adherence to the trial protocol. Any deviations or issues are promptly documented and discussed. Additionally, internal audits of the trial are conducted every 6 months to further ensure compliance and data integrity.

### Plans for communicating important protocol amendments to relevant parties (e.g., trial participants, ethical committees) {25}

Any significant alterations to the protocol that could affect the study’s implementation will be communicated to the stakeholder group and to the University of Siegen’s ethics committee for re-evaluation and approval. Once approval is obtained, the corresponding author will notify the stakeholder group, the primary working group, and the research assistants of the revisions. Participants will also be informed of these changes. Any deviations from the protocol will be fully documented to ensure transparency and proper tracking. Additionally, the updated protocol will be reflected in the clinical trial registry.

### Dissemination plans {31a}

It is planned to publish the results in a peer-reviewed journal. Additionally, abstracts will be submitted to suitable congresses. Participants will be provided a summary of the trial’s results upon request.

## Discussion

The current study aims to examine the potential benefits of incorporating a smoking cessation intervention prior to an outpatient psychotherapy, considering tobacco dependence a significant health concern. While patients with other substance use disorders in Germany are required to achieve abstinence within the initial ten sessions of psychotherapy for the psychotherapy to be covered by health insurance, tobacco smoking remains unaddressed in the field of psychotherapy, despite existing evidence of nicotine’s negative effect on mental health [14, 63] and the potential benefits of smoking cessation for improving mental health symptoms and enhancing emotional and cognitive processes critical for psychotherapy responsiveness. Hence, although existing research emphasizes the favorable impact of smoking cessation on mental health, its practical integration into psychotherapeutic settings remains ambiguous.

Notably, to our knowledge, smoking cessation, specifically prior to psychotherapy in an outpatient psychotherapeutic setting, has not yet been examined experimentally. Although the current state of research suggests that a psychological smoking cessation intervention is effective among smokers with mental disorders [36], it is essential to investigate the underlying mechanisms and conditions that contribute to positive outcomes, particularly in relation to mental health status.

One significant factor to consider is how motivational variables may influence participant engagement and outcomes. In our psychotherapeutic care research, we opted not to restrict participation based on the willingness to quit smoking. Instead, while participants are required to show an interest in changing their smoking behavior, we proactively invited patients to participate in the study. By evaluating participants across various motivational stages using the transtheoretical model of change [51], we aim to assess the impact of these factors on our primary outcomes. This approach allows us to engage a more diverse and representative sample, rather than relying solely on patients’ initial explicit motivation to quit. This will enable us to examine the effects of actively addressing smoking behavior through interventions on smoking behavior, dependence severity, and mental health symptoms. Secondary analyses of motivation and self-efficacy will be performed to detect confounding effects on the primary outcome, but also to explore possible mechanisms of action which might hold significance for future studies, such as ensuring sufficient self-efficacy before the first session. By examining these factors, we can better understand the underlying processes influencing the effectiveness of the intervention and provide valuable insights for optimizing future research and practice.

The potential findings from this study could significantly influence both research and clinical practice. In terms of research, if our results demonstrate positive treatment effects on mental health outcomes, this could warrant further investigation into how smoking cessation prior to psychotherapy affects subsequent therapeutic sessions. Specifically, the findings may reveal previously overlooked impacts of smoking on psychotherapeutic practice, potentially clarifying instances of nonresponse or slow progress in treatment. This insight could lead to a deeper understanding of smoking's role in therapy outcomes and inform more effective, integrated treatment approaches. Future studies should not only examine the immediate effects of smoking cessation on symptoms but also explore its potential to enhance overall treatment effectiveness and contribute to sustained therapeutic success. In terms of clinical practice, In terms of clinical practice, if smoking cessation is shown to enhance mental health outcomes, it could lead to its integration into standard psychotherapeutic protocols, similar to requirements for other substance use disorders. Integrating smoking cessation with standard psychotherapeutic practices could offer a more holistic approach, optimizing patient outcomes and informing future clinical guidelines.

Notwithstanding notable strengths, some limiting factors comprise the generalizability of our findings, which may be constrained by the specific characteristics of our sample population and the setting in which the study will be conducted. The study will be carried out in a single outpatient psychotherapeutic clinic, limiting the diversity of participants and potentially affecting the applicability of our findings to other settings or populations. Nevertheless, the study aims to provide new insights into integrating smoking cessation within psychotherapy, emphasizing its potential role in comprehensive mental health treatment.

## Trial status

The current trial was prospectively registered in the ISRCTN registry for current controlled trials on 01.05.2024 (ISRCTN12859609, protocol version number 1.0). The data collection started in August 2024 and will end by April 2027.

## Data Availability

It is planned to publish the results of the trial in a peer-reviewed journal. Additionally, the final trial dataset will be provided upon request.

## Abbreviations

AAT: Approach-Avoidance-Task
ANOVA: Analysis of variance
APOI: Attitudes toward Psychological Online Interventions
ASSIST: Alcohol, Smoking and Substance Involvement Screening Test
AWMF: Association of the Scientific Medical Societies
BDI-II: Beck Depression Inventory, Revised
BfArM: German Federal Institute for Drugs and Medical Devices
BSCL: Brief Symptom Checklist
CBT: Cognitive‒behavioral therapy
CO: Carbon monoxide
FEVER: University of Rhode Island Change Assessment Scale
FTND: Fagerström Test for Nicotine Dependence
GSE: Generalized Self-Efficacy Scale
ICD-10: International Statistical Classification of Diseases and Related Health Problems
ITT: Intention-to-treat
MTSS: Motivation to Stop Scale
ROC_AUC_: Area under the receiver operating characteristics
SESS: Self-Efficacy Scale for Smoking
SOC: Scale for Stages of Change
TAU: Treatment a usual
WHO: World Health Organization
WHO-5: WHO-5 Well-being Index
WHOQOL-BREF: WHO Quality of Life Brief Questionnaire
